# *Mycobacterium tuberculosis* Rv2882c Protein Induces Activation of Macrophages through TLR4 and Exhibits Vaccine Potential

**DOI:** 10.1371/journal.pone.0164458

**Published:** 2016-10-06

**Authors:** Han-Gyu Choi, Seunga Choi, Yong Woo Back, Hye-Soo Park, Hyun Shik Bae, Chul Hee Choi, Hwa-Jung Kim

**Affiliations:** Department of Microbiology, and Medical Science, College of Medicine, Chungnam National University, Daejeon 301-747, Republic of Korea; Bose Institute, INDIA

## Abstract

Macrophages constitute the first line of defense against *Mycobacterium tuberculosis* and are critical in linking innate and adaptive immunity. Therefore, the identification and characterization of mycobacterial proteins that modulate macrophage function are essential for understanding tuberculosis pathogenesis. In this study, we identified the novel macrophage-activating protein, Rv2882c, from *M*. *tuberculosis* culture filtrate proteins. Recombinant Rv2882c protein activated macrophages to secrete pro-inflammatory cytokines and express co-stimulatory and major histocompatibility complex molecules via Toll-like receptor 4, myeloid differentiation primary response protein 88, and Toll/IL-1 receptor-domain-containing adaptor inducing IFN-beta. Mitogen-activated protein kinases and NF-κB signaling pathways were involved in Rv2882c-induced macrophage activation. Further, Rv2882c-treated macrophages induced expansion of the effector/memory T cell population and Th1 immune responses. In addition, boosting Bacillus Calmette-Guerin vaccination with Rv2882c improved protective efficacy against *M*. *tuberculosis* in our model system. These results suggest that Rv2882c is an antigen that could be used for tuberculosis vaccine development.

## Introduction

Tuberculosis (TB) is a leading cause of human mortality and infectious disease-related morbidity worldwide [1]. The emergence of drug-resistant strains has complicated the control of TB. However, the only available vaccine, *Mycobacterium bovis* Bacillus Calmette-Guerin (BCG), is unable to provide significant protection against pulmonary TB, with the exception of the most severe forms of TB in early childhood [2]. Although various new TB vaccines are in development [3], vaccines that are safe and effective in latently infected individuals and adults are still urgently needed. Therefore, identification and characterization of diverse mycobacterial antigens capable of inducing immunity against *M*. *tuberculosis* (Mtb) will provide a better understanding of host-pathogen interactions and can facilitate the development of effective vaccines.

Macrophages constitute the first line of defense against mycobacteria. They are critical in linking innate and adaptive immunity and serve as the host cell niche that allow Mtb to survive [4]. Upon mycobacterial infection, macrophages recognize, bind, and internalize Mtb. This response initiates a complex process of controlling the intracellular growth of the bacilli, such as secretion of soluble antimicrobial and innate immune mediators. In particular, macrophages activated by Mtb or its components secrete chemokines and cytokines, the most important being tumor necrosis factor-α (TNF-α), cytokines of the interleukin-1 family (IL-1β, IL-18), and IL-12, thereby promoting lymphocyte activation and recruitment, and ultimately inducing granuloma formation [5]. Recognition of mycobacteria or mycobacterial proteins is performed by Toll-like receptors (TLRs), which are expressed mainly on immune cells [6]. After the interaction of specific mycobacterial components with TLRs, signaling pathways are triggered in which the adaptor molecule myeloid differentiation primary response protein 88 (MyD88) plays an important role [7]. In addition, mitogen-activated protein kinases (MAPKs) and NF-κB are activated by the TLR signaling cascade [8]. Through this cascade, mediated by TLR2 or TLR4, various mycobacterial proteins have been reported to induce activation of macrophages or dendritic cells [9–16]. Dendritic cells are widely accepted to be the key cells needed to initiate a T cell response, and macrophages are important for the effector phase of an immune response. Although several mycobacterial proteins that activate macrophages to secrete pro-inflammatory cytokines have been characterized, little is known about the protective role of these mycobacterial proteins in host defense against TB.

In this study, we identified a novel macrophage-activating mycobacterial protein from Mtb culture filtrate proteins (CFPs) by multidimensional fractionation and then investigated its immunoreactivity. We found that a recombinant of this newly identified Rv2882c protein activated macrophages to secrete pro-inflammatory cytokines and to express CD80 and CD86 co-stimulatory molecules and MHC class I/II molecules through TLR4, MyD88, and TRIF. Rv2882c-activated macrophages induced a significant expansion of the effector/memory T cell population. Moreover, Rv2882c exhibited short-term protective efficacy in a BCG prime-boost vaccination in a mouse model.

## Materials and Methods

### Ethics statement

All animal procedures were approved by the Institutional Animal Care and Use Committees of Chungnam National University (Permit Number: CNU-00284). All animal experiments were performed in accordance with Korean Food and Drug Administration (KFDA) guidelines.

### Bacterial strains, animals and cell preparations

Mtb H37Rv (ATCC 27294) and H37Ra (ATCC 25177) were purchased from American Type Culture Collection (ATCC, Manassas, VA). *M*. *bovis* BCG (Tokyo strain) was kindly provided by Korean Institute of Tuberculosis (KIT). All mycobacteria were grown in 7H9 medium supplemented with 0.5% glycerol, 0.05% Tween-80 (Sigma, St. Louis, MO, USA), 10% oleic acid, albumin, dextrose, and catalase (OADC; BD Biosciences, San Jose, CA, USA).

Specific pathogen-free female C57BL/6 mice (6 weeks old) were purchased from Charles River Laboratories (Wilmington, MA), and 5- to 6-week-old C57BL/6J TLR2 knockout (TLR2^-/-^; B6.129-Tlr2tm1Kir/J) and C57BL/10 TLR4 knockout (TLR4^-/-^; C57BL/10ScNJ) mice were purchased from the Jackson Laboratory (Bar Harbor, ME, USA.) The mice were maintained under barrier conditions in a biohazard animal room at the Medical Research Center of Chungnam National University, Daejeon, Korea. The animals were fed a sterile commercial mouse diet with ad libitum access to water under standardized light-controlled conditions (12-h light and 12-h dark periods). The mice were monitored daily, and none of the mice showed any clinical symptoms or illness during this experiment.

BMDMs were flushed through the femurs of C57BL/6 mice with Dulbecco's modified Eagle's medium (DMEM) (Welgene Co., Daegu, Korea). Erythrocytes were lysed by applying RBC lysis buffer (Sigma-Aldrich, St. Louis, MO) for 3 min at room temperature. After washing the cells and preparing a single-cell suspension, total cells were suspended in DMEM containing 10% fetal bovine serum, 50 ng/mL mouse macrophage colony stimulating factor (M-CSF), and 1% antibiotics (Welgene). The cells were then placed in 100-mm plates and incubated for 7 days at 37°C in 5% CO2. The medium was replaced every 3 days during a 7-day incubation.

Lungs were isolated under sterile conditions, cut into 0.5-cm pieces, and agitated in 5 mL cell dissociation buffer (RPMI medium containing 0.1% collagenase type IV (Worthington Biochemical Corporation, NJ, USA), 1 mM CaCl_2_, and 1 mM MgCl_2_) for 15 min at 37°C. Then, the lung cells and aggregates were filtered through a 40-μm cell strainer in Dulbecco’s phosphate-buffered saline (PBS) using a sterile 1-mL syringe. The spleens were mashed on a 40-μm cell strainer in RPMI medium (Welgene). The erythrocytes were lysed using RBC lysis buffer for 2 min at room temperature. High-gradient magnetic-activated cell sorting (MACS) (Miltenyi Biotec, Bergisch Gladbach, Germany) was used to fractionate the T cell subsets. The cells were incubated with anti-mouse CD4 Ab-coated magnetic microbeads (Miltenyi Biotec) and then were positively selected on paramagnetic columns (LS columns; Miltenyi Biotec) according to the manufacturer’s instructions.

### Antibodies and reagents

Recombinant M-CSF was purchased from Peprotech (Rocky Hill, NJ, USA). Fluorescein isothiocyanate (FITC)-annexin V/PI kits were purchased from BD Biosciences (BD Pharmingen, San Jose, CA). LPS from *E*. *coli* O111:B4 was purchased from InvivoGen (San Diego, CA, USA). Endotoxin filter (END-X) and endotoxin removal resin (END-X B15) were acquired from the Associates of Cape Cod (East Falmouth, MA, USA). Anti-phosphorylated ERK1/2 monoclonal Ab, anti-ERK1/2 monoclonal Ab, anti-phosphorylated p38 monoclonal Ab, anti-p38 monoclonal Ab, anti-phosphorylated JNK monoclonal Ab, anti-JNK monoclonal Ab, anti-phosphorylated IκB-α monoclonal Ab, anti-IκB-α monoclonal Ab, and anti-tubulin polyclonal Ab were obtained from Cell Signaling Technology (Danvers, MA, USA). Anti-TLR2, anti-TLR4, and anti-histidine (His) antibodies (Abs) were purchased from Santa Cruz Biotechnology (Santa Cruz, CA, USA). HRP-conjugated anti-mouse IgG Ab and HRP-conjugated anti-rabbit Ab were obtained from Calbiochem (San Diego, CA, USA). Phycoerythrin (PE)-conjugated mAbs directed against IL-10, IL-12p70, CD80, CD86, and MHC class II, and allophycocyanin-conjugated mAb directed against F4/80 were purchased from eBioscience (San Diego, CA, USA). Mouse TNF-α, MCP-1, IL-6, IL-10, IL-12p70, IFN-γ, and IL-2 ELISA kits were obtained from eBioscience.

### Multi-dimensional fractionation of Mtb CFPs

Mtb H37Rv (ATCC 27294) was grown for 6 weeks at 37°C as surface pellicles in Sauton’s medium, and then the CFPs were prepared as previously described [17]. The CFPs were precipitated with 80% ammonium sulfate and then sequentially fractionated by HIC, HAT, and IEC as previously described[18]. The protein bands of interest were identified on CB stained SDS-PAGE gels at the Yonsei Proteomics Research Center (Yonsei University, Seoul, Korea) by LC-ESI/MS as previously described[17].

### Production of recombinant Rv2882c

To produce the recombinant Rv2882c protein, the corresponding gene (*frr*) was amplified by PCR using genomic DNA from Mtb H37Rv (ATCC 27294) as the template and the following primers: forward, 5′-CATATGATTGATGAGGCTCTCTTCGAC-3′ and reverse, 5′-AAGCTTGACCTCCAGCAGCTCGCCTTC-3′. The resulting PCR product was inserted into the pET-22b (+) vector (Novagen, Madison, WI, USA) after digesting both using the *Nde*I and *Hind*III restriction enzymes. The recombinant protein was prepared as previously described [19]. To remove endotoxin contamination from the purified protein, the recombinant protein was incubated with polymyxin B-agarose (Sigma Chemical Co.) for 6 h at 4°C. The amount of residual LPS in the Rv2882c preparation was evaluated using a LAL test kit (Lonza, Basel, Switzerland) according to the manufacturer’s instructions. Purified endotoxin-free Rv2882c was filter sterilized and frozen at −70°C. The purity of the Rv2882c protein was evaluated by CB staining and western blot analysis using an anti-His antibody.

### Cytotoxicity analysis

Rv2882c (10 μg/mL) was added to isolated BMDMs cultured in 12-well plates (0.5 × 10^6^ cells/mL) to investigate the cytotoxic effect of Rv2882c. After 24 h of treatment, the harvested BMDMs were washed using PBS, stained with FITC-Annexin V and PI (BD Biosciences), and then analyzed using a FACSCanto flow cytometer (BD Biosciences).

### Cytokine measurements

Sandwich ELISAs were used to determine the levels of IL-6, MCP-1, TNF-α, IL-12p70, IL-10, IFN-γ, and IL-2 in the culture supernatants as previously described [18]. These cytokine assays were performed as recommended by the antibodies’ manufacturer (eBioscience).

### Analysis of intracellular cytokine and surface molecule expression by flow cytometry

Single-cell suspensions were stimulated with LPS (100 ng/mL) or Rv2882c (10 μg/mL), or left unstimulated, for 12 h in the presence of GolgiPlug (BD Biosciences). The samples were first blocked for 15 min in 10% (vol/vol) normal goat serum and stained at 4°C in flow cytometry buffer (PBS/2% BSA) with APC-conjugated F4/80 antibodies for 30 min at 4°C. Cells stained with the appropriate isotype-matched immunoglobulin were used as negative controls. The cells were fixed and permeabilized using a Cytofix/Cytoperm kit (BD Biosciences) according to the manufacturer’s instructions. Intracellular cytokines were detected using fluorescein-conjugated antibodies (eBiosciences) in a permeabilization buffer. Cell surface staining was performed using specifically labeled fluorescent-conjugated mAbs, and the staining intensity was determined using flow cytometry (FACSCanto, BD Biosciences), after which the data were analyzed using FlowJo data analysis software (BD Biosciences), as recently described [18].

### Pull down assays

BMDMs (1×10^7^) were lysed with lysis buffer (50 mM Tris HCl, pH 8.0; 137 mM NaCl; 1 mM EDTA; 1% (vol/vol) Triton X-100; 10% (vol/vol) glycerol; 1 mM PMSF; 1 μg/mL each of aprotinin, leupeptin, and pepstatin; 1 mM Na3VO4; and 1 mM NaF). Twenty micrograms of the His-tagged Rv2882c protein and cell lysate were mixed and incubated at 4°C for 6 h, and then Ni-NTA agarose beads were added (Qiagen, Chatsworth, CA, USA) according to the manufacturer’s instructions. The beads were mixed with the cell lysate diluted in lysis buffer and rocked for 2 hr at 4°C. After incubation, the mixtures of beads and cell lysates were centrifuged at 150 g for 1 min at 4°C, and then the beads were washed three times with buffer and collected by centrifugation. After removing the supernatant, the beads were boiled in SDS-PAGE sample buffer, and the bound proteins were analyzed by immunoblotting with anti-TLR2, anti-TLR4, and anti-His Abs.

### Immunoblotting analysis

After stimulation with 10 μg/mL Rv2882c, the BMDMs were lysed in 100 μL of lysis buffer. Immunoblotting was conducted as previously described [18]. The epitopes of the target proteins, including MAPKs, were labeled using specific Abs, and the bands were visualized using an ECL Advance kit (GE Healthcare, Little Chalfont, UK).

### Treatment of BMDMs with pharmacological signaling inhibitors

All of the pharmacological inhibitors used in this study were purchased from Calbiochem. Dimethyl sulfoxide (Sigma) was added to the cultures at a final concentration of 0.1% (vol/vol) as a solvent control. The BMDMs were washed using PBS and pretreated with the inhibitors in DMEM medium containing glutamine for 1 h prior to treatment with Rv2882c for 24 h. The inhibitors were used at the following concentrations, as determined by careful titration: U0126 (10 μM), SB203580 (20 μM), SP600125 (10 μM), and Bay11-7082 (5 μM). The viability of BMDMs was assessed using an MTT assay.

### In vitro Mtb growth assay in BMDMs

BMDMs were infected with Mtb at a multiplicity of infection of 1:1 (bacteria to BMDMs) for 4 h. Then, the infected BMDMs were treated with amikacin (200 μg/mL) for 2 h, followed by two washes with PBS. The infected BMDMs were treated with Rv2882c (10 mg/mL) or LPS (100 ng/mL) for 24 h, and then co-cultured with CD4^+^ T cells for 3 days. To determine the bacterial burden, a fraction of the macrophage cultures was immediately lysed using 0.1% saponin. The supernatants and cell lysates were serially diluted and plated on 7H10 agar plates supplemented with 10% OADC enrichment medium to determine the bacterial burden per well.

### BCG boosting vaccine immunization

After the BCG (5 × 10^4^ CFU/0.2 mL/subcutaneous) vaccination, 5 mice per group were subcutaneously immunized on their backs with 10 μg of Ag85B and Rv2882c emulsified with dimethyl dioctadecylammonium bromide (DDA; Sigma-Aldrich) and 25 μg monophosphoryl lipid A (MPL; Sigma-Aldrich) in a total volume of 0.2 mL two times over a 4-week interval. After 6 weeks, the vaccinated mice were infected with Mtb H37Ra (ATCC 25177). Briefly, following anesthetization with a xylazine:zoletil (9:1) mixture, 5 mice per group were intratracheally infected with 50-μL suspensions at infectious dose of 10^6^ CFU of H37Ra per mouse lung.

### Statistical analysis

All experiments were repeated at least three times, with consistent results. The significance of differences between two groups was determined by unpaired Student's *t*-test, and the differences between three or more groups were evaluated with one-way ANOVA followed by Tukey's multiple comparison test using GraphPad Prism (version 4.03) statistical software (GraphPad Software, San Diego, CA, USA). The data in the graphs are expressed as the mean values ± SD; *p < 0.05, **p < 0.01, and ***p < 0.001 were considered statistically significant.

## Results

### Identification of the Rv2882c protein from the Mtb culture filtrates

**Fig 1:**
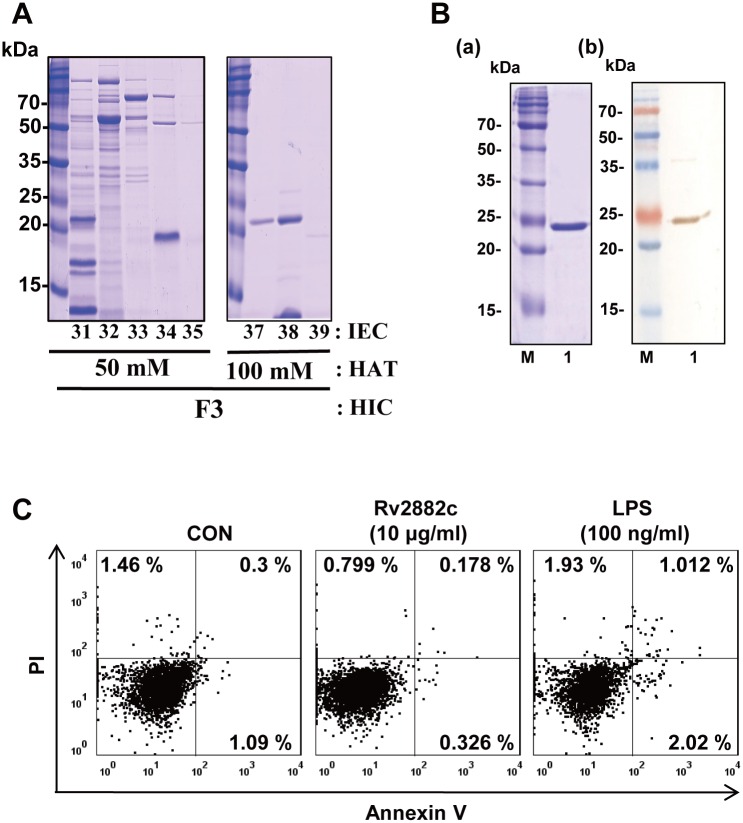
Preparation and cytotoxicity of the recombinant Rv2882c protein. (A) The ammonium sulfate precipitate of the CFPs was fractionated by hydrophobic interaction chromatography (HIC) using Phenyl Sepharose. The primary fractions were divided and concentrated into seven fractions. Each of the primary fractions was further fractionated by hydroxyapatite chromatography (HAT). The eluates were pooled into five to nine fractions based on the protein band pattern and were concentrated. A third fractionation was performed using DEAE ion-exchange chromatography. The proteins were analyzed by SDS-PAGE with Coomassie brilliant blue. (B) Recombinant Rv2882c was produced in BL21 cells and purified using an NTA resin. The purified protein was subjected to (a) SDS-PAGE and (b) western blot analysis using a mouse anti-His Ab. (C) The cytotoxic effect of Rv2882c on BMDMs was analyzed by flow cytometry. BMDMs (1 × 10^5^/well) were stimulated with Rv2882c (10 μg/mL) was added on day 6, and the cultures were harvested 24 h later. The BMDMs were stained with anti-F4/80, annexin V, and PI. The percentage of cells that are positive (annexin V- and PI-stained cells) in each quadrant is indicated. The results are representative of three experiments.

Mtb CFPs were fractionated using multistep chromatography to overcome the limitation of valuable antigen screening due to the complex Mtb antigens. As shown in S1 Fig, an 80% ammonium sulfate precipitate of the CFPs was separated into seven fractions by hydrophobic interaction chromatography (HIC), and then further fractionated by hydroxyapatite chromatography (HAT) and ion-exchange chromatography (IEC). Each individual fraction was tested for its ability to stimulate macrophages to secrete pro-inflammatory cytokines. IEC fraction number 37 from the 100 mM HAT fraction and initial fraction 3 strongly induced IL-12 secretion and appeared as a single band on an SDS-PAGE gel (Fig 1A). This major band was identified as Rv2882c by liquid chromatography-electrospray ionization-tandem mass spectrometry (LC-ESI/MS). A recombinant Rv2882c protein was purified from *Escherichia coli*, and its purity was confirmed by SDS-PAGE. The purified protein had a molecular mass of approximately 23 kDa and reacted with anti-His antibody (Ab) (Fig 1B). Endotoxin content was measured by a limulus amoebocyte lysate (LAL) assay, and only the protein lots with extremely low endotoxin content (<0.2 EU/mL) were used in subsequent experiments (S2 Fig). To determine whether cytotoxicity in response to Rv2882c affected macrophage activation and cytokine secretion, the viability of bone marrow-derived macrophages (BMDMs) treated with 10 μg/mL Rv2882c for 24 h was assessed by annexin V and propidium iodide (PI) staining. As shown in Fig 1C, Rv2882c was not toxic to BMDMs.

### Rv2882c activates macrophage to secrete pro-inflammatory cytokines and to increase the expression of co-stimulatory and MHC molecules

**Fig 2:**
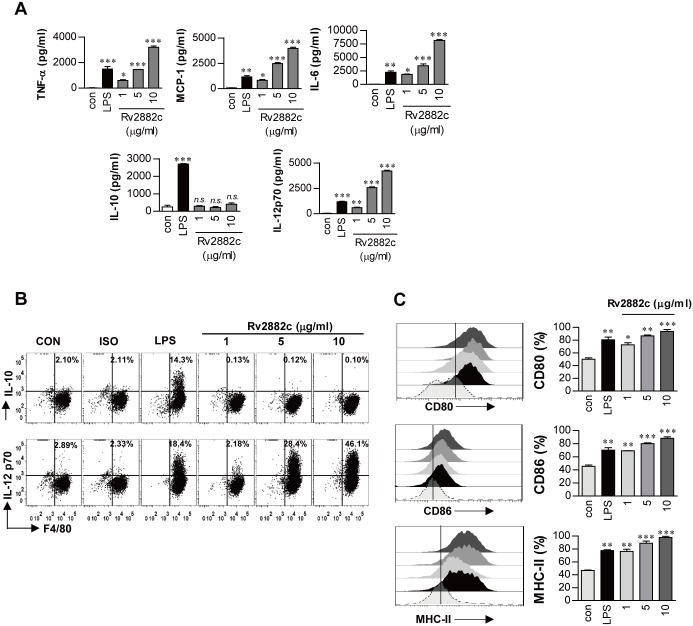
Rv2882c induced BMDM activation. (A) BMDMs (1 × 10^5^/well) were stimulated with 100 ng/mL LPS or 1, 5, or 10 μg/mL Rv2882c for 24 h. (A) Quantities of TNF-α, MCP-1, IL-6, IL-10, and IL-12p70 in the culture supernatant were determined by ELISA. All data are expressed as the mean values ± SD (*n* = 3). Significance levels (*p < 0.05, **p < 0.01 or ***p < 0.001, determined by one-way ANOVA test) of the differences between the treatment data and the control data are indicated; treatments that were not significantly different are indicated by *n*.*s*. (B) Dot plots of the intracellular IL-12p70 and IL-10 concentrations in the F4/80^+^ BMDMs. The percentage of cells that are positive is shown in each panel. (C) The BMDMs were prepared as described in (A) and analyzed for the expression of surface markers using flow cytometry. The cells were gated on the F4/80^+^ BMDMs. The BMDMs were stained with anti-CD80, anti-CD86, and anti-MHC class II antibodies. The percentage of cells that are positive is shown in each panel. The bar graphs depict the mean values ± SD (*n* = 3). The levels of significance (*p < 0.05, **p < 0.01 or ***p < 0.001, determined by one-way ANOVA test) of the differences between the treatment data and the control data are indicated.

To confirm whether recombinant Rv2882c induced macrophage activation and stimulated pro-inflammatory cytokine production in macrophages, cytokine levels in the culture supernatants of BMDMs treated with Rv2882c at 1, 5 or 10 μg/mL for 24 h were determined. LPS was used as a positive control. Rv2882c significantly increased the production of TNF-α, IL-6, IL-12, and MCP-1, but not IL-10, in a dose-dependent manner (Fig 2A). In contrast, LPS only induced IL-10 production significantly, in comparison with untreated cells or Rv2882c-treated cells. Intracellular staining for IL-12 and IL-10 also indicated that Rv2882c significantly induced IL-12, but not IL-10, production, and that LPS stimulated significant production of only IL-10 in BMDMs (Fig 2B). Next, we investigated whether Rv2882c modulated the expression of macrophage surface molecules, which are important for the function of macrophages as antigen presenting cells and phagocytes. Flow cytometry showed that Rv2882c significantly upregulated the expression of CD80, CD86, and MHC class II molecules in a dose-dependent manner, when compared to the expression levels in untreated cells. This effect was comparable to that of LPS used as a positive control (Fig 2C). Although LPS was removed from the purified recombinant protein, we tested whether Rv2882c-induced macrophage activation could be attributed to LPS contamination. Both proteinase K digestion and heat denaturation of the Rv2882c protein abrogated TNF-α, IL-6, and MCP-1 production (S2 Fig), and heat-denatured Rv2882c protein significantly decreased the cell surface expression of CD86 and MHC-II (S3 Fig). Furthermore, pre-incubation of the BMDMs with polymyxin B did not suppress Rv2882c-induced cytokine production, but did reduce the stimulatory effects of LPS (S2 Fig), indicating that the biological activity of Rv2882c was not the result of LPS contamination. These results suggest that Rv2882c can effectively induce macrophage activation, promoting its functional role as an APC.

### Rv2882c induces macrophage activation through the TLR4 pathway

**Fig 3:**
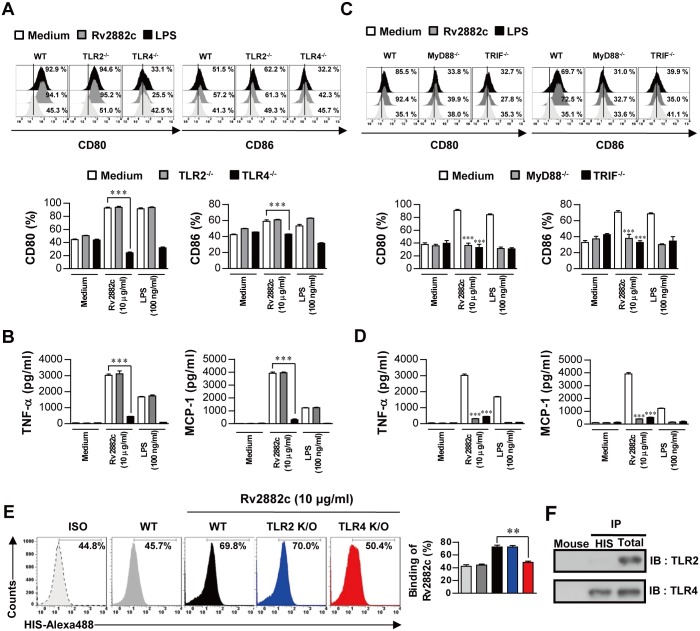
Rv2882c induced BMDM activation via TLR4-dependent MAPKs and NF-κB. (A, B) Bar graphs showing the level of CD80 or CD86 expression on Rv2882c-treated F4/80^+^-gated BMDMs derived from WT, TLR2^−/−^, and TLR4^−/−^mice. BMDMs (1 × 10^5^/well) derived from WT, TLR2^−/−^, and TLR4^−/−^mice were treated with Rv2882c (10 μg/mL) for 24 h. The percentage of cells that are positive is shown in each panel. The bar graphs show the mean percentage ± SEM of each surface molecule on F4/80^+^ cells across three independent experiments. The supernatants were determined by enzyme-linked immunosorbent assay (ELISA). All data are expressed as the mean values ± SD (*n* = 3); ***p < 0.001 = significance of treatment values compared to those of Rv2882c-treated WT BMDMs. (C, D) Bar graphs showing the level of CD80 or CD86 expression on Rv2882c-treated F4/80^+^-gated BMDMs derived from WT, MyD88^−/−^, and TRIF^−/−^mice. BMDMs derived from WT, MyD88^−/−^, and TRIF^−/−^mice were treated with Rv2882c (10 μg/mL) for 24 h. The percentage of cells that are positive is shown in each panel. The bar graphs show the mean percentage ± SEM for each surface molecule on F4/80^+^ cells across three independent experiments. The supernatants were evaluated using an ELISA. All data are expressed as the mean values ± SD (*n* = 3); ***p < 0.001 = significance of treatment values compared to Rv2882c-treated WT BMDM values. (E) BMDMs derived from WT, TLR2^−/−^, and TLR4^−/−^mice were treated with Rv2882c (10 μg/mL) for 1 h and were stained with a FITC-conjugated anti-His monoclonal antibody (mAb). The percentage of cells that are positive is shown in each panel. The bar graphs depict the mean values ± SD (*n* = 3); ***p < 0.001 = significance of Rv2882c-treated TLR4^−/−^BMDM values compared to those of Rv2882c-treated WT BMDM. (F) Immunoprecipitation (IP) with anti-His and immunoblotting (IB) with anti-TLR2 or -TLR4 antibodies. BMDMs (2 × 10^6^/well) were treated with Rv2882c (10 μg/mL) for 6 h. The cells were harvested, and cell lysates were used for immunoprecipitation with anti-mouse IgG, anti-His, anti-TLR2, or anti-TLR4. Then, proteins were visualized by immunoblotting with anti-TLR2 or anti-TLR4 Abs. The totals shown represent the mean total cell lysates (input).

Several mycobacterial proteins have been reported to activate macrophages through TLR2- and/or TLR4-dependent signaling pathways [16, 20–22]. To determine whether Rv2882c-induced macrophage activation is TLR-dependent, we prepared BMDMs from C57BL/6J wild-type (WT), TLR2^-/-^, and TLR4^-/-^ mice, and then treated them with Rv2882c for 24 h. The expression of surface molecules such as CD80 and CD86 and production of pro-inflammatory cytokines were significantly reduced in the Rv2882c-treated BMDMs from the TLR4^-/-^ mice, but not from the WT or TLR2^-/-^ mice (Fig 3A and 3B). TLRs are critical in provoking innate immune responses by initiating signaling cascades through Toll/IL-1 receptor (TIR) domain-containing adaptors such as MyD88 and TRIF [23]. MyD88 is common to all TLRs, whereas TRIF is essential for TLR4-mediated activation of the MyD88-independent signaling pathway [23]. As expected, the Rv2882c-induced expression of surface molecules and production of pro-inflammatory cytokines was significantly reduced in the BMDMs from the MyD88^-/-^ and TRIF^-/-^ mice compared to those from the WT mice (Fig 3C and 3D). Next, using an Alexa488-conjugated anti-His polyclonal antibody, we examined whether Rv2882c could bind to macrophage surfaces via TLR4. Flow cytometry revealed that Rv2882c bound to the surface of the WT and TLR2^-/-^ macrophages, but not to that of the TLR4^-/-^ macrophages (Fig 3E). We performed an immunoprecipitation analysis with anti-TLR2 or anti-TLR4 antibody and an anti-His antibody to confirm the interaction between Rv2882c and TLR4. Rv2882c bound to TLR4, but not to TLR2 (Fig 3F). These findings clearly show that Rv2882c induces macrophage activation through TLR4 to increase the expression of cell surface molecules and the production of pro-inflammatory cytokines.

### Rv2882c induces macrophage activation through mitogen-activated protein kinases (MAPKs) and the NF-κB pathway

**Fig 4:**
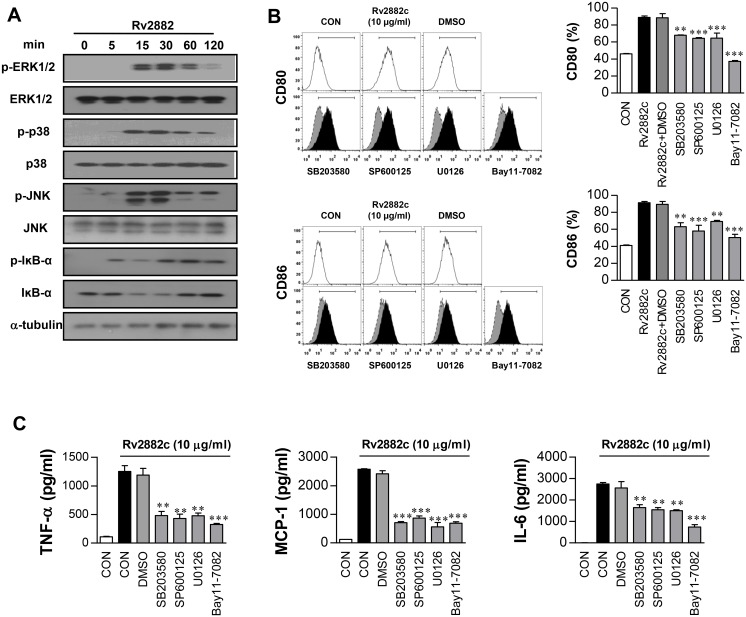
BMDM activation triggered by Rv2882c resulted in the activation of MAPKs and NF-κB. (A) Protein expression over time in BMDMs (1 × 10^6^/well) treated with 10 μg/mL Rv2882c. The cell lysates were subjected to SDS-PAGE and immunoblotted using Abs specific to phospho-p38 (p-p38), p38, phospho-ERK1/2 (p-ERK1/2), ERK1/2, phospho-IκB- (p-IκB-), IκB-, phospho-JNK (p-JNK), and JNK. α-tubulin was used as the loading control for the cytosolic fractions. (B) BMDMs were treated with pharmacological inhibitors of p38 (SB203580, 20 μM), ERK1/2 (U0126, 10 μM), and NF-κB (Bay11-7082, 5 μM) or with DMSO (vehicle control) for 1 h prior to treatment with 10 μg/mL Rv2882c for 24 h. (C) The amounts of TNF-α, IL-6, and MCP-1 in the culture media were determined by ELISA. The data shown are the mean values ± SD (*n* = 3). One representative plot out of three independent experiments is shown; ***p < 0.001 = a significant difference, as determined by unpaired Student’s *t*-test, between the Rv2882c-treated BMDM values and those of the BMDMs treated with Rv2882c and pharmacological inhibitors.

MAPKs and NF-κB are critical factors that induce cellular immune responses, including the production of pro-inflammatory cytokines in macrophages [24]. Therefore, we examined the activation of MAPKs and NF-κB in response to Rv2882c. Immunoblotting to analyze the phosphorylation profiles of ERK1/2, p38, JNK, and NF-κB in BMDMs revealed that Rv2882c triggered the phosphorylation of MAPKs, including p38, ERK1/2, and JNK in macrophages (Fig 4A). In addition, Rv2882c also induced the phosphorylation and degradation of IκB-α and significant translocation of p65 from the cytosol to the nucleus. Polymyxin B treatment did not affect Rv2882c-induced phosphorylation of MAPKs and NF-κB, but did inhibit LPS-induced phosphorylation (S3 Fig).

Next, the importance of MAPK and NF-κB activity for Rv2882c-induced activation of macrophages was examined using highly specific kinase inhibitors. Rv2882c-induced pro-inflammatory cytokine production was significantly reduced by the pharmacological inhibitors tested, and expression of co-stimulatory molecules on the surface of macrophages was significantly blocked by these inhibitors (Fig 4B and 4C). These results suggest that the MAPK and NF-κB signaling pathways are essential for Rv2882c-induced macrophage activation.

### Rv2882c induces the expansion of the effector/memory T cell population

**Fig 5:**
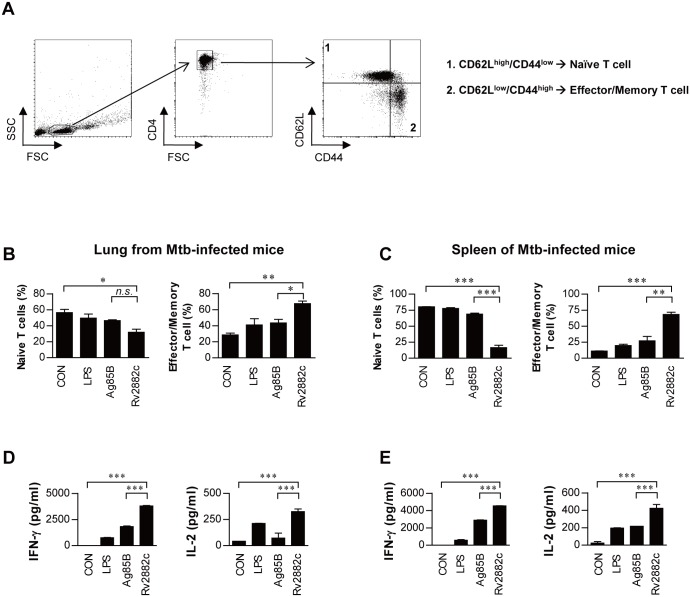
*Ex vivo* Rv2882c stimulation induced Ag-specific memory T cell expansion in spleen and lung cells from Mtb-infected mice. The cells were prepared as described the Materials and Methods. (A) Gating strategy to assess intracellular cytokines and transcription factors. All samples were stained for surface molecules and gated based on forward scatter (FSC) and side scatter (SSC). The T cells were gated from the lymphocyte gate by FSC vs. SSC based on surface expression patterns of CD4^+^ T cells. Using the CD4^+^ T cell gate, cells specific staining for CD62L and CD44 are shown in stimulated spleen or lung cells. The BMDMs (1 × 10^5^/well) were treated with Rv2882c (10 μg/mL) or LPS (100 ng/mL) for 24 h. Next, media or CD4^+^ T cells (1 × 10^6^/well) from the lungs (B, D) or spleens (C, E) of Mtb-infected mice were added to each well and incubated for 3 days. (B and C) The numbers of naïve CD4^+^ T cells (CD4^+^CD44^-^CD62L^+^) and effector/memory CD4^+^ T cells (CD4^+^CD44^+^CD62L^-^) were determined by flow cytometry. (D and E) The levels of IFN-γ and IL-2 were determined by ELISA. The data shown are the mean values ± SD (n = 15); *p < 0.05, **p < 0.01 or ***p < 0.001 = a significant difference between Rv2882c-treated and untreated or Rv2882c-treated and Ag85B-treated cells, as determined by one-way ANOVA. Treatments with no significant effect are indicated by *n*.*s*.

To assess whether macrophages activated by Rv2882c specifically induce the effector/memory T cell population in Mtb-infected mice, we analyzed the surface expression levels of CD62L and CD44 on CD4^+^ T cells using flow cytometry. Naïve T cells have previously been reported to express a CD62L^high^CD44^low^ phenotype, whereas effector/memory T cells exhibit a CD62L^low^CD44^high^ phenotype [25]. CD4^+^ T cells were isolated via MACS from spleen or lung cell suspensions derived from Mtb H37Ra-infected WT mice at 4 weeks post-infection were co-cultured with Rv2882c-treated BMDMs derived from uninfected mice for 3 days. As shown in Fig 5A, the CD4 labeled cells were gated to assess the molecules on T cell surfaces by FACS analysis. Antigen 85B (Ag85B) is a potent vaccine constituent, and it was used as a control antigen. Rv2882c-treated macrophages from the lungs and spleens of mice induced a significant expansion of the effector/memory (CD62L^low^/CD44^high^) T cell population compared to LPS- or Ag85B-treated macrophages, and the population of naïve (CD62L^high^/CD44^low^) T cells in the spleen and lungs was significantly decreased in co-cultures with Rv2882c-treated macrophages (Fig 5B and 5C). In addition, IFN-γ and IL-2 levels were subjected to ELISA to assess the production of cytokines that are related to the differentiation of Th1 cells into effector and memory cells [26, 27]. Similar to the response pattern of the effector/memory T cells, the lung and spleen T cells that were co-cultured with Rv2882c-treated macrophages produced significantly higher levels of IFN-γ and IL-2 compared to the LPS- or Ag85-treated macrophages (Fig 5D and 5E). IL-4 and IL-5 was not detected in any conditions. These data show that Rv2882c can induce the development and expansion of effector/memory T cells.

### Boosting BCG with Rv2882c improves protective efficacy of the vaccine

First, we tested Rv2882c for its immunoreactivity in terms of recognition by T lymphocytes from Mtb-infected mice. As shown in S4 Fig, the Rv2882c protein led to a significant increase in IFN-γ production in comparison with the Ag85B protein in the lungs and spleen from BCG-infected mice or H37Ra-infected mice. In addition, induction of IFN-γ production and secretion by the Rv2882c protein was stronger in the lungs and spleen of H37Ra-infected mice than in those organs of BCG-infected mice. Therefore, Rv2882c has greater antigenicity for recognition by T lymphocytes during H37Ra infection than during BCG infection. On the basis of these results, we can say that Rv2882c has a potential as a TB vaccine candidate.

**Fig 6:**
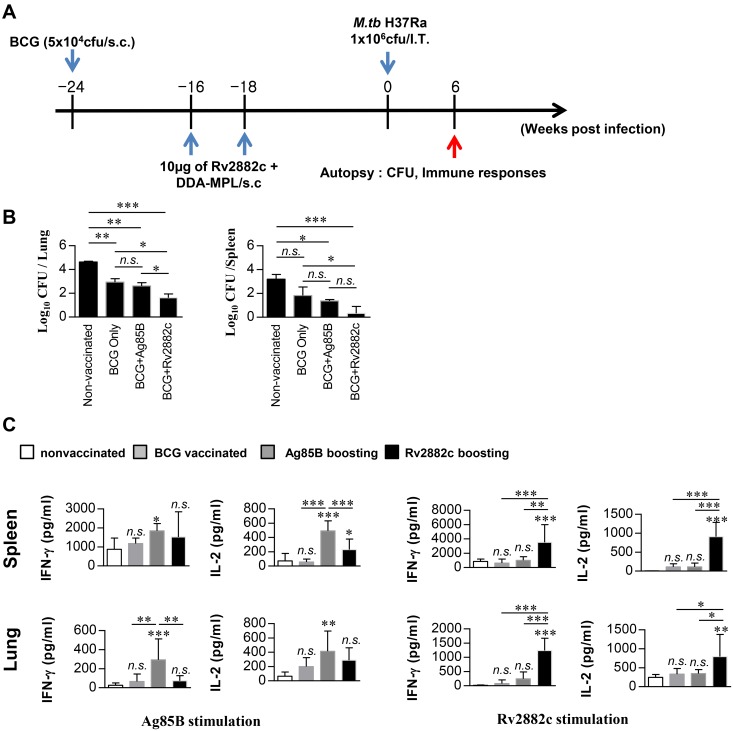
Rv2882c boosting enhanced the BCG protective immune response in an *in vivo* model. The infection procedure is described in the Materials and Methods. Six weeks after challenge, animals’ spleens and lungs were harvested and the number of bacteria (CFUs) per organ was counted. (A) Schedule for BCG immunization, Rv2882c boosting, and Mtb challenge. (B) Bacterial loads in the lungs and spleens of each group are represented as the mean (± SEM) log_10_ CFUs/organ (n = 5); *p < 0.05; **p < 0.01; ***P < 0.001 vs. the infection-only group. (C) Spleen and lung cells were harvested from mice in each group, and the cytokine levels were analyzed by ELISA. The lung and spleen cells (10^6^/well) were treated with Ag85B (10 μg/mL) or Rv2882c (10 μg/mL) for 3 days. These cells were obtained from each group of vaccinated mice. The data are shown as mean ± SD (n = 15); *p < 0.05, **p < 0.01, or ***p < 0.001: a significant difference between Rv2882c- or Ag85B-restimulated nonvaccination and vaccination groups, as determined by one-way ANOVA. Treatments without significant effects are labeled with *n*.*s*.

To determine whether Rv2882c has the potential to be used as a vaccine, we examined the level of protection against an Mtb H37Ra-challenge following a BCG boost with Rv2882c compared to protection from BCG immunization alone. Mtb H37Rv and H37Ra strains were derived from the same parental strain, but differ in their virulence in experimental animals [28]. It is easier to use the avirulent H37Ra strain in experiments, as there is no risk of infection. To determine whether it was possible to test vaccine efficacy using a mouse model and the H37Ra strain, we first investigated the persistence of this strain in mice. The growth of Mtb H37Ra in the mouse lung peaked at 3 weeks after intratracheal infection, and their numbers maintained (within 1 log) in the lungs and spleens of mice until 26 weeks (S5 Fig), indicating that Mtb H37Ra remained viable and could be used in screening vaccine candidates. As shown in Fig 6A, all mice were vaccinated and challenged intratracheally with Mtb H37Ra. The mycobacterial burdens in the lungs and spleens were determined at 6 weeks after challenge. Ag85B was included as a control antigen. BCG vaccine significantly decreased the bacterial load in the lungs (−2.02 log_10_) and spleen (-2.1 log_10_) compared to that of the non-vaccinated group (Fig 6B). The bacterial loads in the antigen-boosted groups were also significantly decreased relative to those in the non-vaccinated group. The Ag85B-boosted mice had slightly lower bacterial counts than the BCG-vaccinated mice, but this difference was not significant. Interestingly the Rv2882c-boosted mice had significantly lower bacterial counts than the BCG-vaccinated mice (*P* < 0.05). In lungs, the Rv2882c-boosted mice had significantly lower bacterial counts than the Ag85B-boosted mice did. Th1-mediated immune responses play an important role in protective immunity against Mtb [29]. At 6 weeks after Mtb challenge, splenocytes or lung cells were stimulated *in vitro* with Ag85B or Rv2882c, and the cytokines produced were determined by ELISA. The stimulating antigens specifically induced cytokine production in cells from the corresponding antigen-boosted mice (Fig 6C). Activation of IL-2 and IFN-γ production by Rv2882c was significantly stronger in lung cells and splenocytes from Rv2882c-boosted mice than from mice immunized with BCG alone or from Ag85B-boosted mice. These results suggest that boosting BCG with Rv2882c elicits a protective effect and a Th1 cytokine response in our model.

## Discussion

Macrophages express TLRs and phagocytic receptors that play crucial roles in the recognition of and response to pathogens, and are essential in the initiation of an innate immune response [30]. Many mycobacterial components are potent activators of macrophages and act as ligands to stimulate an innate immune response [31, 32]. Ligation of TLRs by mycobacteria or their components initiates a signaling cascade in macrophages and dendritic cells that culminates in the production of pro-inflammatory cytokines and chemokines [33] that are crucial to eliciting an adaptive immune response against the pathogen [34]. In this study, we showed that a newly identified Mtb protein, Rv2882c, strongly activates macrophages to produce pro-inflammatory cytokines and express co-stimulatory and MHC molecules via the TLR4 pathway.

Previously, we found that fractionating Mtb CFPs by multistep chromatography and determining their immunoreactivity was a powerful approach to identifying proteins from the complex Mtb antigen system [35]. In this study, we identified a novel macrophage-activating protein, Rv2882c, through multidimensional fractionation of Mtb CFPs. Rv2882c is a ribosome recycling factor [36] that is found in the membrane fraction and culture filtrate of Mtb H37Rv [37–39]. However, the functional role of Rv2882c during mycobacterial infection is largely unknown, and its immunological properties have not been demonstrated.

We found that levels of the pro-inflammatory cytokines TNF-α, IL-6, and MCP-1 were increased in BMDMs stimulated with Rv2882c protein. TNF-α, a critical pro-inflammatory cytokine, plays an important role in host protective immune responses against mycobacterial infection [40]. In addition, MCP-1 contributes substantially to host anti-mycobacterial inflammatory responses [41]. Rv2882c also significantly increased the secretion of IL-12p70, a key player in the host defense against mycobacteria, whereas the secretion of IL-10, an immune-regulatory cytokine, was not induced. IL-12 is a critical factor that drives the generation of IFN-γ-producing T cells, which are essential for controlling an Mtb infection. The protective role of the IFN-γ/IL-12 immune axis is well established [42, 43]. Taken together, our data suggest that the Rv2882c protein activates macrophages and drives a Th1-type immune response.

Macrophages are principal regulators of immunity by phagocytosis, presentation of antigens to T cells, and enhancement of the effector T cell responses. Highly polymorphic MHC class I and II molecules and co-stimulatory molecules are involved in these responses [44, 45]. In the current study, Rv2882c stimulated macrophages, thereby augmenting the expression of CD80, CD86, and MHC class II molecules. These data suggest that Rv2882c enhances the ability of macrophages to activate T cells for protective immunity against mycobacteria. Pozzi et al. showed that macrophages do prime naïve T cells to proliferate and mature into both effector and memory cells by direct presentation [46]. In this study, Rv2882c-activated macrophages induced a significant expansion of the effector/memory T cell population and production of IFN-γ and IL-2 in co-cultures with spleen or lung cells from Mtb-infected mice, relative to the effect of Ag85B-activated macrophages from the same source. As expected, LPS-activated macrophages did not induce an effector/memory T cell response.

In the present study, by immunoprecipitation assay and experiments using TLR4 or TLR2 knockout mice, we showed that Rv2882c activation of macrophages is mediated by TLR4, MyD88, and TRIF, and that Rv2882c increases the expression of co-stimulatory molecules and pro-inflammatory cytokines through the NF-κB and MAPK signaling pathways. Several mycobacterial components have been reported to induce activation of macrophages or dendritic cells via TLR2- or TLR4-mediated pathways [9]; i.e., TLR2 for most PE/PPE proteins [10–12] and 19-kDa lipoprotein [13], and TLR4 for heat-shock protein 65 [14], RpfE [15], and Rv0652 [16]. TLRs lead to diverse responses, including activation of dendritic cells to drive Th1 [12, 15, 18] or Th2 immune responses [47], as well as activation of macrophages to secrete anti-inflammatory cytokine IL-10 [11] or pro-inflammatory cytokines [10, 16, 48], indicating that an individual antigen can have different effects in macrophages and dendritic cells. Other studies have shown that mycobacteria or their components trigger intracellular signaling pathways that involve MAPKs and NF-κB [31, 49, 50]. The similarity of the responses induced by Rv2882c and LPS and the signaling of Rv2882c chiefly through TLR4 suggested the possibility of LPS contamination in the protein preparation. However, several lines of evidence indicate that signaling via TLR4 and activation of macrophages was attributable to Rv2882c rather than LPS contamination. MyD88 plays a central role in initiating the innate immune response to Mtb [51]. Although the role of TLR4 in host defense against tuberculosis is controversial, studies have reported that innate recognition by TLR4 may play a protective role in tuberculosis. One study revealed increased susceptibility to Mtb infection in C3H/HeJ mice, which have nonfunctional TLR4, in keeping with enhanced mycobacterial outgrowth and an increased mortality [52, 53]. Another study also showed that C3H/HeJ mice are more susceptible to pulmonary tuberculosis, judging by reduced survival and enhanced mycobacterial outgrowth [54]. Therefore, we can hypothesize that TLR4-mediated immune responses triggered by the Rv2882c protein may contribute to protective immunity against Mtb infection. In addition to MyD88, TLR4 can induce intracellular signaling through a second pathway, which is mediated by the adaptor molecule TRIF. Here, we also showed that Rv2882c induced production of TNF-α and MCP-1 in through MyD88 and TRIF.

Recently, a 19-kDa lipoprotein was found to exert anti-mycobacterial activity through the induction of autophagy in human macrophages [55]. However, this lipoprotein inhibits MHC-II expression [13] and IFN-γ-regulated MHC-II antigen processing [56] in macrophages following prolonged exposure to the protein; it also inhibits CD4^+^ T cell activation. PPE18 activates macrophage that then secrete IL-10 [11], and Rv1917c induces maturation of dendritic cells to drive a Th2 immune response [47]. Therefore, these proteins may be not suitable for inclusion in a TB vaccine. In contrast, our data indicated that the Rv2882c protein (in comparison with the Ag85B protein) led to a significant increase in IFN-γ production in the lungs and spleen of Mtb-infected mice. Rv2882c has higher antigenicity for recognition by T lymphocytes during H37Ra infection than during BCG infection. Consequently, in this study, Rv2882c induced macrophage activation, resulting in the secretion of pro-inflammatory cytokines, expansion of the effector/memory T cell population, and induction of a Th1 immune response, and suggesting that this protein might be a valuable vaccine antigen. Therefore, we tested the protective efficacy of Rv2882c as a BCG vaccine booster in an established model using Mtb H37Ra. Because the efficacy of BCG vaccine alone or with Ag85-boosting has been assessed in our test system, we believed it could be used to test whether a protein has vaccine potential. We demonstrated that boosting BCG with Rv2882c improved the protective efficacy of the vaccine vs. BCG alone or BCG-prime followed by Ag85B-boost. However, the limitations of our study were that we determined only the short-term protective effect of Rv2882c and that vaccine efficacy was assessed only against an avirulent Mtb strain. Therefore, further studies on the efficacy of vaccination with this protein and on the protective mechanism of Rv2882c against Mtb H37Rv are being conducted. Collectively, the data presented in this report suggest that the novel immunostimulatory antigen Rv2882c should be a candidate for the rational design of an effective TB vaccine.

## Supporting Information

S1 Fig Identification of the Mtb protein Rv2882c. The ammonium sulfate precipitate of the CFPs was fractionated by hydrophobic interaction chromatography (HIC) using Phenyl Sepharose. The primary fractions were divided and concentrated into seven fractions. Each of the primary fractions was further fractionated by hydroxyapatite chromatography (HAT). The eluates were pooled into five to nine fractions based on the protein band pattern and were concentrated. A third fractionation was performed using DEAE ion-exchange chromatography.
(TIF)

S2 Fig Confirmation of decontamination of purified Rv2882c from endotoxin by the LAL assay and ELISA. (A) The amount of residual LPS in the RpfE preparation was estimated using the Limulus amoebocyte lysate (LAL) test according to the manufacturer’s instructions. To ensure that Rv2882c-induced BMDM (1 × 10^5^/well) activation was not due to endotoxin contamination in the protein preparation, Rv2882c (10 μg/mL) was (B) denatured by heating for 1 h at 100°C, (C) digested with Proteinase K (10 μg/mL) for 1 h at 37°C, or (D) pretreated with polymyxin B (50 μg/mL) for 1 h prior to stimulating the BMDM cultures. After 24 h, the quantities of TNF-α and IL-6 in the culture medium were measured by ELISA. All data are expressed as the mean values ± SD (*n* = 3); ***p < 0.001 = a significant difference compared to the Rv2882c-treated BMDMs, as determined by unpaired Student’s t-test. Treatments with no significant effect are indicated by *n*.*s*.
(TIF)

S3 Fig Confirmation of endotoxin decontamination of the purified Rv2882c by FACs and western. (A) BMDMs (1 × 10^5^/well) were stimulated with LPS (100 ng/mL), Rv2882c (10 μg/mL), heat denaturated LPS (100 ng/mL) or heat denaturated Rv2882c (10 μg/mL) for 24 h. The BMDMs analyzed for the expression of surface markers using flow cytometry. The cells were gated on the F4/80^+^ BMDMs. The BMDMs were stained with anti-CD86 and anti-MHC class II antibodies. The percentage of cells that are positive is shown in each panel. (B) BMDMs were treated with Rv2882c (10 μg/mL), Rv2882c (10 μg/mL) mixed with polymyxin B, LPS (100 ng/mL), and LPS (100 ng/mL) mixed with polymyxin B in time course. The mixture was prepared by reacting Rv2882c (10 μg/mL) or LPS (100 ng/mL) with polymyxin B (50 μg/mL) for 1 h prior before treatment. Cell lysates were subjected to SDS-PAGE and immunoblotted using Abs specific to phospho-p38 (p-p38), p38, phospho-ERK1/2 (p-ERK1/2), ERK1/2, phospho-JNK (p-JNK), JNK, phospho-IκB- (p-IκB-), and IκB-. α-tubulin was used as the loading control for cytosolic fractions.
(TIF)

S4 Fig Antigenicity of the Rv2882c protein. The cells were prepared as described in *Materials and Methods*. The lung and spleen cells (5 × 10^6^/well) were treated with Rv2882c (10 μg/mL) or Ag85B (10 μg/mL) for 5 days. The levels of IFN-γ in the culture supernatants were determined by an ELISA. The data are shown as mean ± SD (n = 5); *p < 0.05, **p < 0.01, or ***p < 0.001: a significant difference between treated and untreated groups, as determined by one-way ANOVA. Treatments without a significant effect are indicated by *n*.*s*.
(TIF)

S5 Fig Long-term bacterial growth curve in the lungs and spleens of mice infected with avirulent Mtb. The mice were intratracheally infected with 10^6^ CFUs of Mtb H37Ra. The bacterial loads in the lungs and spleens were determined at 3, 6, 10, 15, 20, and 26 weeks.
(TIF)
