# Supplementary figures and images for: Mycobacterium tuberculosis Rv2882c Protein Induces Activation of Macrophages through TLR4 and Exhibits Vaccine Potential
Source: PLoS One. 2016 Oct 6;11(10):e0164458. doi: 10.1371/journal.pone.0164458 (PMC5053528; doi:10.1371/journal.pone.0164458)

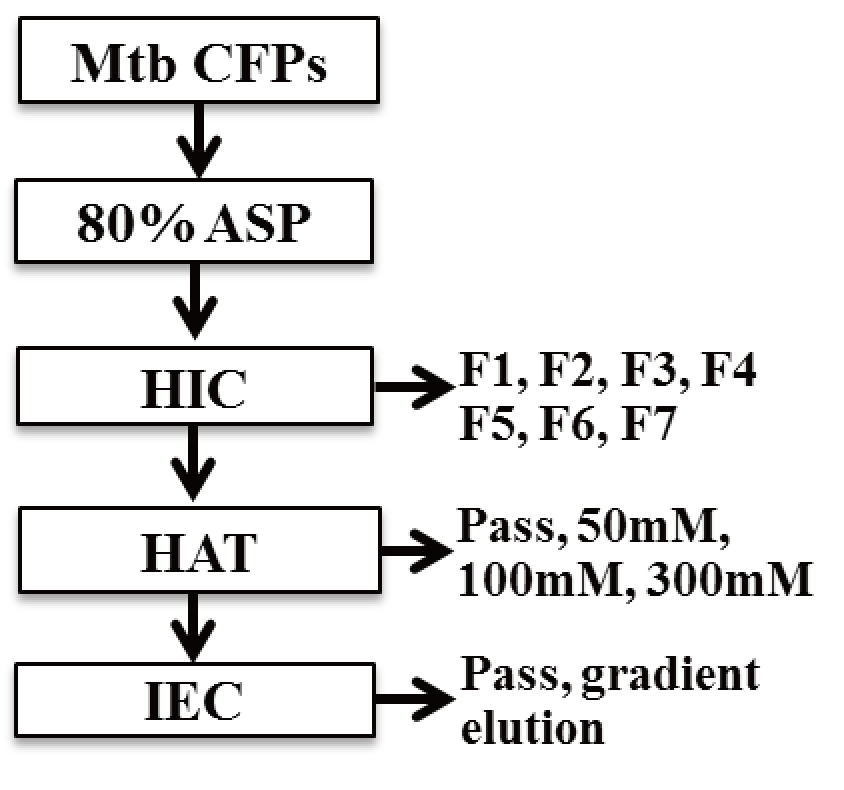

Supplement: S1 Fig — The ammonium sulfate precipitate of the CFPs was fractionated by hydrophobic interaction chromatography (HIC) using Phenyl Sepharose. The primary fractions were divided and concentrated into seven fractions. Each of the primary fractions was further fractionated by hydroxyapatite chromatography (HAT). The eluates were pooled into five to nine fractions based on the protein band pattern and were concentrated. A third fractionation was performed using DEAE ion-exchange chromatography. (TIF) [file pone.0164458.s001.tif]

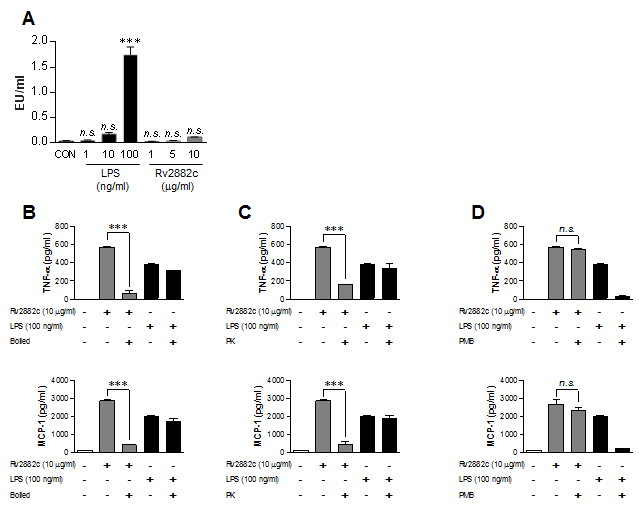

Supplement: S2 Fig — (A) The amount of residual LPS in the RpfE preparation was estimated using the Limulus amoebocyte lysate (LAL) test according to the manufacturer’s instructions. To ensure that Rv2882c-induced BMDM (1 × 10^5^/well) activation was not due to endotoxin contamination in the protein preparation, Rv2882c (10 μg/mL) was (B) denatured by heating for 1 h at 100°C, (C) digested with Proteinase K (10 μg/mL) for 1 h at 37°C, or (D) pretreated with polymyxin B (50 μg/mL) for 1 h prior to stimulating the BMDM cultures. After 24 h, the quantities of TNF-α and IL-6 in the culture medium were measured by ELISA. All data are expressed as the mean values ± SD (n = 3); ***p < 0.001 = a significant difference compared to the Rv2882c-treated BMDMs, as determined by unpaired Student’s t-test. Treatments with no significant effect are indicated by n.s. (TIF) [file pone.0164458.s002.tif]

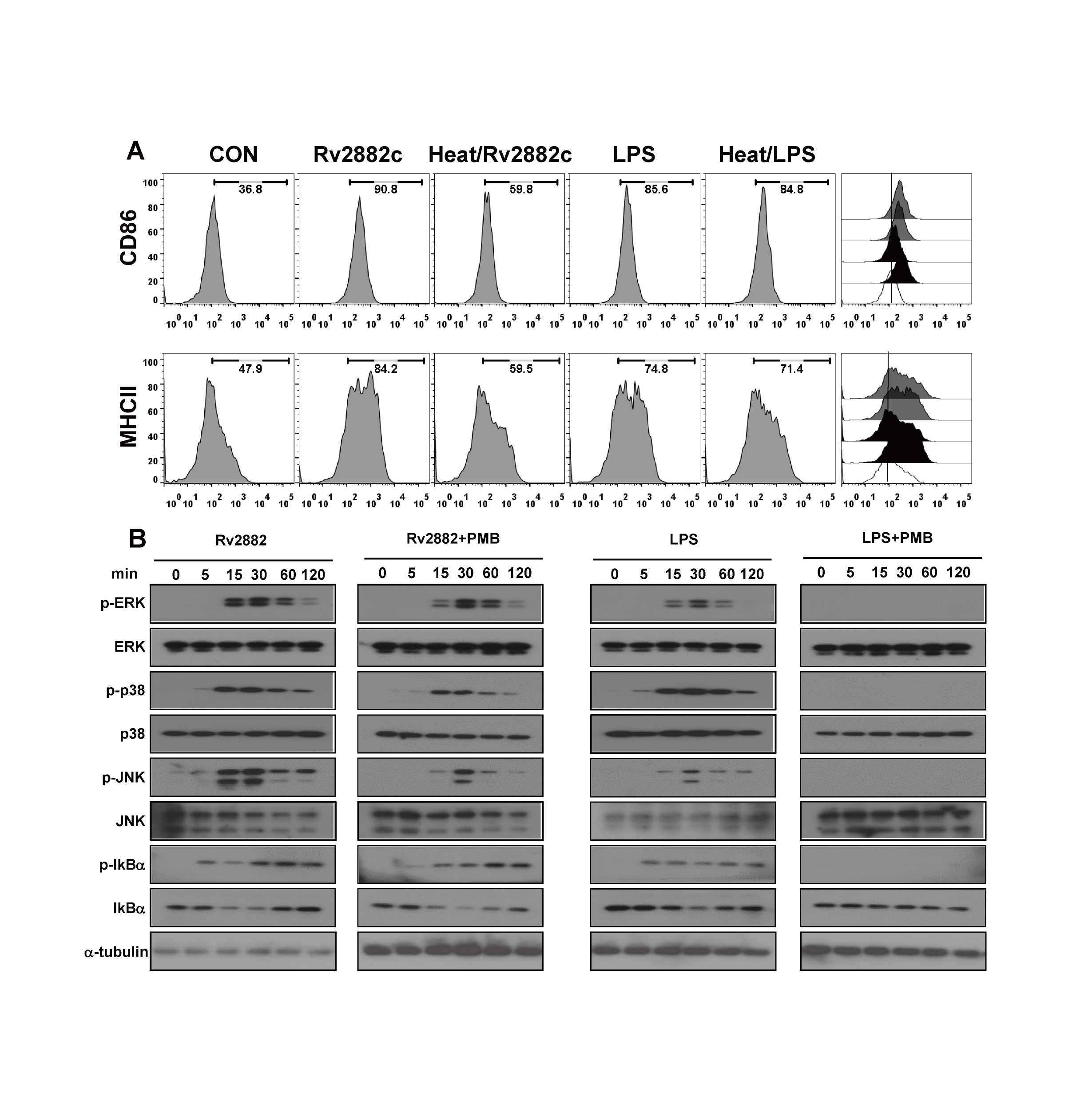

Supplement: S3 Fig — (A) BMDMs (1 × 10^5^/well) were stimulated with LPS (100 ng/mL), Rv2882c (10 μg/mL), heat denaturated LPS (100 ng/mL) or heat denaturated Rv2882c (10 μg/mL) for 24 h. The BMDMs analyzed for the expression of surface markers using flow cytometry. The cells were gated on the F4/80+ BMDMs. The BMDMs were stained with anti-CD86 and anti-MHC class II antibodies. The percentage of cells that are positive is shown in each panel. (B) BMDMs were treated with Rv2882c (10 μg/mL), Rv2882c (10 μg/mL) mixed with polymyxin B, LPS (100 ng/mL), and LPS (100 ng/mL) mixed with polymyxin B in time course. The mixture was prepared by reacting Rv2882c (10 μg/mL) or LPS (100 ng/mL) with polymyxin B (50 μg/mL) for 1 h prior before treatment. Cell lysates were subjected to SDS-PAGE and immunoblotted using Abs specific to phospho-p38 (p-p38), p38, phospho-ERK1/2 (p-ERK1/2), ERK1/2, phospho-JNK (p-JNK), JNK, phospho-IκB- (p-IκB-), and IκB-. α-tubulin was used as the loading control for cytosolic fractions. (TIF) [file pone.0164458.s003.tif]

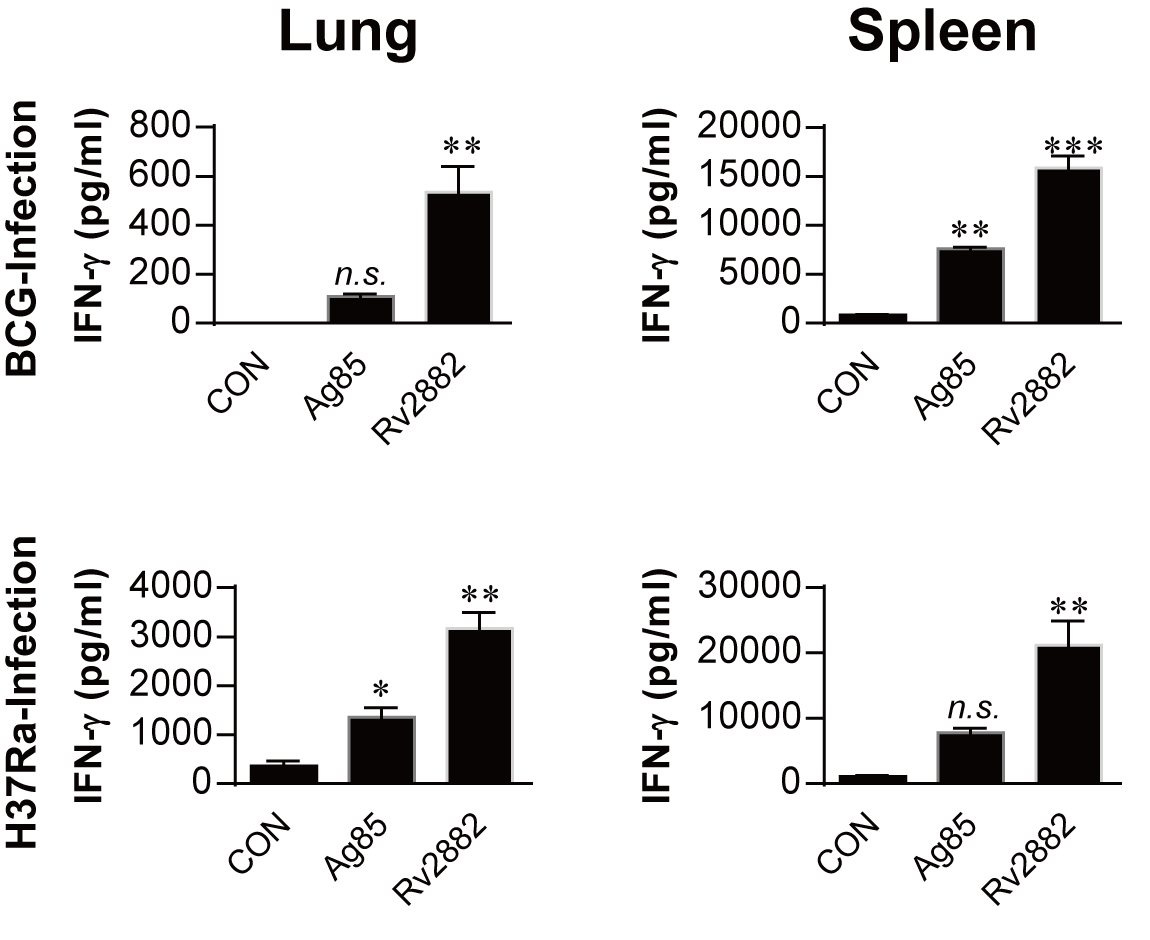

Supplement: S4 Fig — The cells were prepared as described in Materials and Methods. The lung and spleen cells (5 × 10^6^/well) were treated with Rv2882c (10 μg/mL) or Ag85B (10 μg/mL) for 5 days. The levels of IFN-γ in the culture supernatants were determined by an ELISA. The data are shown as mean ± SD (n = 5); *p < 0.05, **p < 0.01, or ***p < 0.001: a significant difference between treated and untreated groups, as determined by one-way ANOVA. Treatments without a significant effect are indicated by n.s. (TIF) [file pone.0164458.s004.tif]

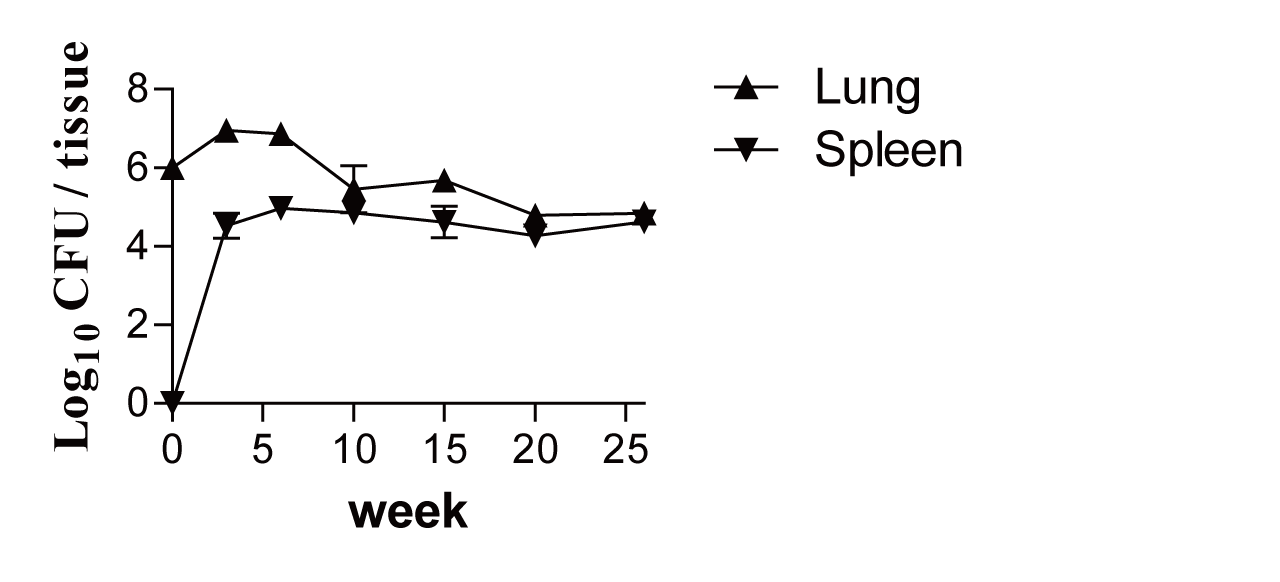

Supplement: S5 Fig — The mice were intratracheally infected with 106 CFUs of Mtb H37Ra. The bacterial loads in the lungs and spleens were determined at 3, 6, 10, 15, 20, and 26 weeks. (TIF) [file pone.0164458.s005.tif]
